# Day Dreams of a Doctor

**Published:** 1898-09

**Authors:** 


Day Dreams of a Doctor. By C. Barlow, M. D. Duodecimo, pp. 251. Buffalo, N. Y.: The Peter Paul Book Company. 1898.

Dr. Barlow has not written a book which will take a high place in literature. He is not an Oliver Wendell Holmes, or a Weir Mitchell, but he has written a book which will interest the physician in that it sets forth more or less accurately the universal experience of the medical man. It will be read, however, with even greater pleasure by the patient than by his physician.

The author draws exact, yet not too technical, two pictures of the work of the surgeon, the bacteriologist, the alienist and other medical specialists, not forgetting the general practitioner. His views on patent medicines and charlatanism generally, which are those of the medical profession at large, are expressed without rancor, yet in such a way as to expose to the layman who reads the folly of all quackery. The book, indeed, is an excellent little work on hygiene, so sugar-coated that the reader will hardly notice that he is taking a pill. One would accuse the author of irony in the chapter on woman were it not for the thread of romance running through the reveries in which the lady doctor figures as heroine, meeting at last the fate which, alas ! so often overtakes the woman physician in real life.

The binding of the book is neat, the paper and typography excellent, but the illustrations are atrocious. M. J. F.

